# How to generate and test hypotheses about colour: insights from half a century of guppy research

**DOI:** 10.1098/rspb.2022.2492

**Published:** 2023-06-14

**Authors:** Darrell J. Kemp, David N. Reznick, Jeffrey Arendt, Cedric van den Berg, John A. Endler

**Affiliations:** ^1^ Evolutionary and Behavioural Ecology Research Group, School of Natural Sciences, Macquarie University, Sydney, New South Wales 2109, Australia; ^2^ Department of Evolution, Ecology and Organismal Biology, University of California, Riverside, CA 92521, USA; ^3^ School of Biological Sciences, University of Bristol, Bristol BS81TQ, UK; ^4^ Centre for Integrative Ecology, School of Life and Environmental Sciences, Deakin University, Waurn Ponds, Victoria 3216, Australia

**Keywords:** colour patterns, colour pattern evolution, colour pattern analysis, colour pattern perception, avoiding analytic errors

## Abstract

Coloration facilitates evolutionary investigations in nature because the interaction between genotype, phenotype and environment is relatively accessible. In a landmark set of studies, Endler addressed this complexity by demonstrating that the evolution of male Trinidadian guppy coloration is shaped by the local balance between selection for mate attractiveness versus crypsis. This became a textbook paradigm for how antagonistic selective pressures may determine evolutionary trajectories in nature. However, recent studies have challenged the generality of this paradigm. Here, we respond to these challenges by reviewing five important yet underappreciated factors that contribute to colour pattern evolution: (i) among-population variation in female preference and correlated variation in male coloration, (ii) differences in how predators versus conspecifics view males, (iii) biased assessment of pigmentary versus structural coloration, (iv) the importance of accounting for multi-species predator communities, and (v) the importance of considering the multivariate genetic architecture and multivariate context of selection and how sexual selection encourages polymorphic divergence. We elaborate these issues using two challenging papers. Our purpose is not to criticize but to point out the potential pitfalls in colour research and to emphasize the depth of consideration necessary for testing evolutionary hypotheses using complex multi-trait phenotypes such as guppy colour patterns.

## 1. Introduction

Complex coloration can facilitate evolutionary investigations in nature because the interaction between genotype, phenotype and environment is relatively accessible. Humans and other species differ in their visual abilities and perception; consequently, biologists invariably experience things differently than the species they study [1–3]. This difference imposes the challenge of how to represent visual phenomena in the terms that matter to natural viewers' perception [2,3] and how to formulate and test appropriate hypotheses [4]. A clearly defined research question is of paramount importance. However, for research to be successful, it must explicitly account for which viewers are involved, their relative abundance, their visual systems, their perception under viewing environmental conditions and any behaviours that may characterize how such phenotypes function [2–6]. The conceptual and practical challenges of colour-based research require detailed consideration of how animals view the world around them.

Here, we summarize some of the pitfalls associated with developing and testing hypotheses concerning non-human visual perception and function, describe the factors affecting coloration and use it to encourage broader and more complete colour pattern investigations. We explore these issues by tracing efforts to understand the evolution of ornamental coloration in wild guppies, *Poecilia reticulata* Peters. Guppies offer a rich medium for discussion because a great deal is known about their visual system, behavioural ecology and evolution [7–14]. The literature devoted to the study of guppy coloration also echoes the development of tools for quantifying colour patterns [2,3,5,11], including efforts to apply the latest colour analytics [15,16]. This extensive history of sensory-oriented guppy research presents a timely narrative for maintaining biological realism in the face of potentially transformative advances in analytical capacity.

## 2. The guppy colour evolution paradigm

**Figure 1.**
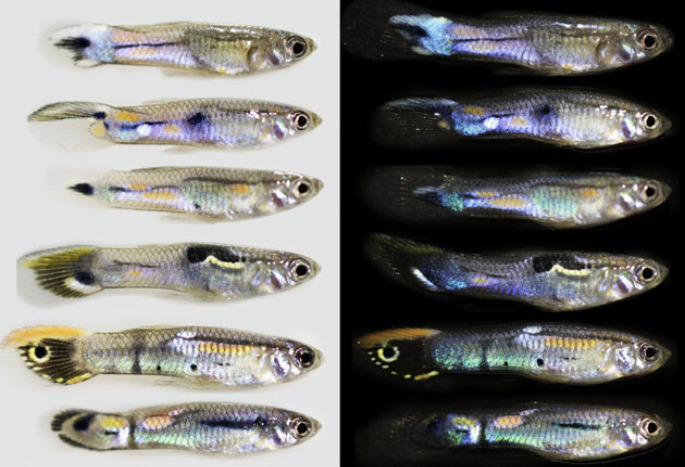
Six male guppies photographed against light and dark backgrounds. Males in the wild are likely to be viewed against both background types and all gradations in-between (and frequently against light/dark mosaic backgrounds [20]). Each background type enhances different features of the coloration: light backgrounds accentuate pigment-based orange and black markings, whereas dark backgrounds accentuate structural coloration. Biases against each background are especially prevalent across the caudal peduncle because the tissue substrate becomes more transparent towards the posterior of the fish. The caudal fin membrane is entirely transparent unless pigmented, which demands a light background to adequately display colour. These guppies are laboratory-reared descendants of wild females sampled from the Trinidad Guanapo drainage and are photographed as described in [14].

Guppies are small freshwater fish native to northeast South America and adjacent islands, where they occupy shallow, clear-water rainforest streams with visually diverse backgrounds comprising multi-coloured gravel, rocks and leaf litter [2]. This species has proven popular for studying animal coloration because males possess extensively polymorphic colour patterns [2,13,17,18]. These patterns consist of discrete pigment-based orange, yellow and black spots, less discrete but temporally dynamic melanic markings [19], called ‘fuzzy black’ [2] and structural colours. The latter markings appear to the human viewer as bright flashes of violet, purple, blue, teal, green and silver, which often grade into each other across the flank and with changing angles of view (figure 1). Guppies and members of their potential viewing audience, such as predatory *Macrobrachium* prawns, perceive a greater range of hues because they can perceive the strong UV reflectance of some colour patches [21–25]. Males bear these complex colour patterns as a consequence of female mating preferences [26]. Guppies also naturally exist with a variety of visually orienting predators, which would otherwise be expected to penalize such conspicuousness [2,27]. Consequently, male guppy coloration is the product of a trade-off between sexual selection, favouring mate attractiveness, versus natural selection, favouring crypsis [2,28,29].

The studies by Endler of male guppy coloration in the late 1970s [2,28,29] produced a textbook paradigm of experimental evolution in the wild. Briefly, evolution was manipulated in two ways. First, Endler transplanted guppies from a downstream predator-rich locality on the Aripo River to an upstream site that excluded virtually all predators, including the most dangerous visually orienting species *Crenicichla frenata* (formerly *Crenicichla alta*). The experimental population experienced a reduction in predation risk, which gave rise to a situation where female preference for male ornamentation prevailed. Endler replicated this experiment in replicate artificial streams in which he manipulated predation risk in both directions by having streams with no predators, reduced predation (only the killifish *Rivulus hartii* present) or high predation (*C. frenata* present) (fig. 1 in [28]). Both experiments revealed that added predation risk caused reduced male ornamentation, while reduced risk caused increased ornamentation. Changes in response to reduced predation risk were most evident via increases in the number and/or size of structurally coloured blue, silver and bronze spots and increases in the size of pigmentary black and orange spots. Structural colours are highly relevant to predation risk because they generate conspicuous flashes of colour visible from a distance, even under low light of the rainforest floor [2,29]. Larger spots are also riskier because they are more likely to exceed predators' chromatic and achromatic visual acuity thresholds, meaning that guppies can be detected from greater distances and under lower light intensities. These results were particularly compelling because they corresponded closely with how male guppy phenotypes vary across locations throughout Trinidad and Venezuela that had these same differences in predation risk [2].

Although investigations of guppy coloration in both natural and experimental populations have since supported this paradigm (e.g. [14,24,25]), recent work has questioned its generality. Two studies [15,16] are particularly notable in this regard. These studies demonstrated new analyses based upon new methods which go beyond the counts and sizes of discrete colour patches, as pioneered by Endler [2,28]. Yong *et al.* [15] applied several components of the quantitative colour pattern analysis (QCPA) framework [30]. This application of QCPA focused on the visual saliency of boundaries (edges between adjacent colour patches) [30]. Valvo *et al.* [16] characterized colour at homologous locations across the organism using their colourmesh tool. Both studies present serious attempts at objectively capturing the spatio-chromatic detail of complex colour patterns, although Valvo *et al.* [16] do not account for sensory aspects of coloration. Both studies addressed whether or not guppy colour phenotypes evolve along consistent trajectories (parallel evolution) under equivalent predation intensities. Both studies defined evolutionary trajectories as vectors in multi-trait space joining the data centroids (multivariate means) for high predation populations to those of low predation populations within the same river. They then tested if the direction of vectors representing differences between high and low predation communities from different rivers was dissimilar to each other. Both authors concluded that the vectors that represent the differences between high and low predation communities in different rivers are not ‘too similar’. They therefore do not support parallel evolution of male coloration across high versus low predation environments. Both studies use these findings to argue that adaptation of the guppy colour phenotype may be neither replicable nor predictable—thereby apparently contradicting the very basis of Endler's paradigm. However, the work by Endler never mentioned or considered the paradigm of parallel evolution with respect to colour patterns, only the responses to sexual and natural selection [2,28,29].

These two studies [15,16] are similar to others (e.g. [31]) in seeking to use male guppy coloration to test the concept of parallel evolution [32]. Here, we point out fundamental problems common across these studies and how these ultimately limit the scope and applicability of their conclusions. One systemic problem is that both studies share and propagate an emerging misunderstanding of what is meant by evolutionary parallelism because they equate parallelism with adaptive evolution. Specifically, both studies measure parallelism by estimating the vector's angle that joins the ancestral to descendant population means. Bolnick *et al*. [32], who promote this geometric interpretation of parallelism, define parallelism as ‘the evolution of similar phenotypes or genotypes in multiple independent populations, in response to similar selection pressures, from similar initial conditions' (p. 302). They also argue that parallelism should be measured in degrees, rather than as a present-absent phenomenon, such ‘that parallel evolution is best viewed as an extreme end of a quantitative continuum of (non)parallel evolution’ (p. 302). Stated differently, to not be ‘too similar’ does not mean that the populations being compared do not display the evolution of similar adaptive traits. One reason is that imperfect or no parallelism can still be consistent with the adaptive evolution of phenotypic similarity in response to similar selection pressures [31]. A second reason is that the same endpoint can be attained by convergent evolution, which [32] defines as ‘the evolution of similar phenotypes or genotypes in multiple populations, in response to similar selection pressures, from different initial conditions' (p. 302). In fact, there is little reason to expect that complex multi-trait phenotypes such as colour patterns should evolve in parallel even when subject to similar overall intensities of selection. Parallelism is not expected when considering polymorphic traits affected by sensory, behavioural and community ecology, sexual selection, evolutionary genetics and multivariate trait evolution; in fact these predict divergence [33,34].

We discuss five main points that collectively illustrate why the hypothesis of evolutionary parallelism is essentially a ‘straw man’, first as a summary and then in detail. We couch our argument in relation to the studies of Yong *et al.* and Valvo *et al.* However, our purpose is not to criticize but to point out the potential pitfalls in colour research and to underscore the depth of consideration needed to test evolutionary hypotheses using complex multi-trait phenotypes. These points apply to any study of coloration:

(1) *Variation in selection*. Given that guppy coloration is conceptualized as a balance between opposing multivariate vectors of sexual and natural selection, population-level contrasts must consider the potential for variation in all sources and targets of selection. The phenotypic target of guppy female choice varies within and among rivers and populations [35,36], as it does in other animals [37]. Differences or changes in predation across populations may therefore see sexual selection favour many possible routes to increased visual conspicuousness [35,38]. Moreover, the complex and spatially variable visual backgrounds in these streams mean that there are a large number of ways of being equally visible, even within a population [2,3]. This could easily promote varied evolutionary trajectories even under constant predation risk.
(2) *Visual acuity and motion*. The distance from which colour phenotypes are seen and the observer's visual acuity will determine the most salient features. Predators detect male guppies from distances that generally preclude their ability to resolve the intricacy of colour patterns *per se*, whereas females appraise courting males at close range [11,39]. This difference in viewing distance is vital in considering how coloration may be influenced by natural versus sexual selection [2]. Motion blur further modifies the appearance of a colour pattern, sometimes profoundly, depending upon motion direction [40,41].
(3) *Structural colour visibility*. Structural colours are often highly visible over long distances and, therefore, particularly relevant to predation risk [2]. Since they flash during courtship, they are possibly more visible under lower light levels than pigment and melanin-based colours. Dedicated measurement protocols are required to estimate these visual features appropriately.
(4) *Viewer sensory properties*. Sensory-based analyses will only yield accurate conclusions and predictions if such analyses include appropriate visual parameters, including effects of perception and eye functions [4,42]. For heterospecific viewers such as predators, this requires that relevant species are correctly identified and that their vision be characterized [11]. Further, if multiple species of predators are present, the relative risk posed by each species should ideally be weighted to derive a composite risk estimate of how prey colour patterns are likely to experience natural selection (as in [2,11]).
(5) *Multivariate selection and genetic response*. It is unrealistic to assume that selection should consistently generate similar changes across all colour pattern component traits, especially when they are polymorphic (multiple forms within a population). There are problems at three levels: individual trait function, phenotypic selection and genetic response. Each trait should have its own array of natural and sexual selection effects as well as potential evolutionary influences modulated by multivariate genetic architecture. This is made more complex by the very complex guppy visual backgrounds and because sexual selection can lead to very different evolutionary trajectories even under identical starting conditions and genetics. Consequently, covariances across the phenotypic selection of variance-covariance matrix should not be assumed to be high and positive but to range anywhere between −1 and 1. Similarly, we must not assume that the genetic covariances are all positive. Consequently, the estimated balance between sexual selection and natural selection for crypsis will depend upon which traits are included in the estimation of colour phenotype and how they are selectively and genetically correlated.

### (a) Variation in selection

Endler's (1980) classic guppy evolution experiments have held great appeal because they illustrate a fundamental theoretical tenet: levels of sexual ornament expression should be balanced against naturally selected viability costs [43]. For guppies, this suggests that selection for net lifetime fitness should favour increasingly ‘colourful’ male phenotypes up until the point where the benefits of sexual attractiveness become offset by the predation risk. By experimentally reducing predation intensity in the wild, Endler [28] shifted the local balance between sexual and natural selection, thereby freeing male colour ornamentation to evolve greater conspicuousness. Sexual selection should drive evolutionary change because females judge mate attractiveness relative to all males available in their population. Relative rather than absolute preferences in sexual selection provide the potential for idiosyncratic or unique evolutionary trajectories that may contribute to population divergence.

The guppy evolution paradigm has since been generalized to predict that populations subject to relatively low predation risk should be more colourful than those under greater risk [23,44,45]. This proposition is reasonable, but only in the broadest sense. It does not mean that all low predation populations should necessarily evolve increased colourfulness in the same way. There is enormous complexity in the potential colour phenotypes/traits that may prove attractive to females. Even within a given population, the visually diverse nature of guppy visual backgrounds presents countless different ways that a male colour pattern could achieve the same degree of conspicuousness [2]. This diversity is compounded by variation among individual females in what they find most attractive [36], including their potential penchant for novelty [46–49]. It is also well known that female preference varies among guppy populations. [35,38,45,50]. Endler & Houde [35,38] demonstrated substantial variation in the target(s) of female preference and male colour patterns across 11 populations from nine different Trinidad rivers. They also found that how colour phenotypes were enhanced in low predation environments differed among rivers and did so in ways commensurate with local female preferences. Were these phenotypes represented as vectors, this result would be directly analogous to a demonstration of non-parallel evolution. The guppy coloration system is polygenic and hence very permissive of variation in appearance and hence allows population divergence shaped by the different combinations of visual backgrounds, lighting conditions [20] and female preferences [35,50] within and among localities. This permissiveness is in fact one of the explanations for their extensive polymorphism [2].

**Figure 2.**
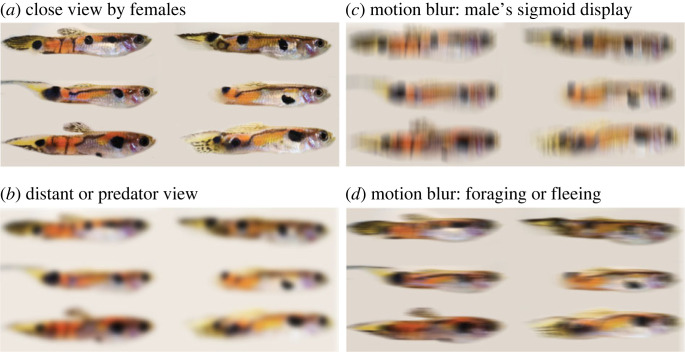
Illustrations showing the potential blurring effects of acuity and motion. Defocus and motion blur were done using MATLAB functions fspecial (for filter definition) and imfilter (to apply the filter to the image). Structural colours show weakly in this photograph; see electronic supplementary material, figures S1 and S2 for effects on structural colours and more details. (*a*) Six guppies seen at short distance (as in typical guppy courtship). (*b*) The same guppies seen at a distance (as by a predator). (*c*) Motion blur during the sigmoid courtship display in which the body is rapidly rotated along the body long axis, blurring vertically. This also has a strong effect on the structural colours because they only reflect strongly in certain directions, causing a strong flicker. (*d*) Motion blur when foraging or fleeing a predator, blurring horizontally. This kind of motion results in a lot less structural colour flicker because the long axis is not rotated very much, if at all. Note the loss of colour and luminance contrast due to acuity limits and note the very different appearances due to motion in different directions. Similar effects occur for structural colours (electronic supplementary material, figures S1 and S2). Guppies and their predators gather very different visual information.

The variation in how guppies adapt to low predation environments has also been demonstrated via transplantation experiments conducted in different rivers. The evolution of male phenotypes following transplantation from predator-rich to previously guppy-free, predator-poor environments has been shown to differ among rivers in ways that align with differences in local female preference [25]. In the Aripo river, males from transplanted populations evolved larger pigmentary and structural coloured markings (fig. 3 in [28]). By contrast, the same experiment conducted in the El Cedro River saw males evolve smaller pigmentary spots coupled with greater areal coverage and reflectance intensity of structural colour (see figs 2 and 3 in [25]; also [14]). These outcomes imply that differences among rivers in how sexual selection manifests will cause divergent vectors of colour evolution. Divergence among populations experiencing similar selective conditions is also expected in the relevant views of both sexual selection and parallel evolution. The Fisher process is unpredictable because the traits under selection can evolve in different directions from the same starting point under identical conditions and genetics, even if the traits are controlled by single loci [51], but guppy coloration is controlled by multiple genes [2]. Parallel evolution [32] also includes a range of patterns between strong (little within regime variation) and no parallel evolution. This continuum is expected among traits and populations, so we should not expect to see ‘too similar’ vectors of change in different populations, especially under sexual selection.

### (b) Visual acuity and motion

Efforts to infer how particular viewers may select colour patterns have considered spectral sensitivity more often than visual acuity [52]. The perception of colour patterns, and therefore their overall visibility and that of its colour pattern elements, likely varies with viewing distance [2,52]. This is because spatial resolving power (acuity)—a product of both visual acuity and viewing distance—determines the extent to which a viewer can appreciate the full detail of a colour pattern and, therefore, its degree of visual contrast [2,39]. Acuity is profoundly relevant for appraising how colour patterns could appear to conspecifics compared to predators [2,30,53].

Predators detect prey from greater distances than conspecifics observe each other during courtship. Endler estimated predator detection and attack distances for guppies based on direct observations in natural streams [11]. Predators detected guppies at distances ranging from five (*Rivulus*, *Macrobrachium*) to twenty times (*Crenicichla*), the distance that female guppies typically view courting males (approx. 20 mm). This difference in viewing range means that fine-scale features of male colour pattern, such as small spots, lines and colour patch boundaries, are far more likely to have salience to courted females than to predators [2,39]. Likewise, the boundaries between otherwise highly contrasting features of the colour pattern are likely to become increasingly blurred with increasing viewing distance and decreasing visual acuity (figure 2*a,b*). It is therefore critical to account for known predator attack ranges (i.e. retina to guppy distances [11]) and visual acuity to determine how colour patterns will be perceived and subsequently selected [2,52].

Motion affects the appearance of colour patterns. If movement is faster than the reaction time of photoreceptors then visual acuity decreases along the axis of motion [54] (figure 2). Although we do not know the flicker-fusion rate for guppies (and most fish), rotation around the body's long axis during the sigmoid guppy courtship display [19] is so fast that it is difficult to film even at 1000 frames per second (J.A.E. 2017, unpublished data). Rotation can result in vertical motion blur leaving fine detail (figure 2*c*). Movement along the body's long axis during foraging or predator escape can lead to a different pattern (figure 2*d*). Spots elongated in the direction of movement are less subject to motion blur than when motion is perpendicular to their elongation. Long patches may also make it difficult for a predator to track the guppy, as in striped snakes [2]. Bright colours in bars can have significantly reduced colour contrast when motion blended, as in coral snakes and their mimics [40]. Consequently, the same colour pattern can appear very different to guppies and predators, depending upon how and when the guppy moves and the visual abilities of each predator (figure 2). We need to know more about the effects of motion blur relative to colour pattern geometry.

Predator spatial acuity is highly relevant to approaches like the work of Yong *et al.* [15] who focused on colour patch boundaries to analyse the visibility/detectability of colour patterns to different viewers, including acuity. Recent work in guppies revealed that boundary strength (a measure of contrast between adjacent colour patches) explained 34–70% of the variation in female mating decisions [41]. By contrast individual boundary contrast explained at best only 6–8% of predator attack success in the triggerfish (*Rhinecanthus aculeatus*) [53]. Troscianko *et al.* [55] found weak predictive power of individual boundary-related traits for the human detection of prey stimuli against a natural background. Although this might suggest that boundaries are important for sexual selection but much less important for predation, these studies differ in which traits were measured, how success was measured and how individual traits were statistically assessed. The ecologically and evolutionarily relevance of these effects is unclear and warrants further behavioural investigation.

Although correlations between colour pattern statistics and predator behaviour can be statistically significant, this does not necessarily indicate biological significance. Moreover, relying heavily on colour patch adjacency and boundary strength by itself, no matter the viewing context or visual properties of a colour pattern, does not best represent how predators detect prey in all contexts, even if it works for mate choice in a specific context in another study. This is a possible explanation for why the analysis by Yong *et al.* of guppy colour pattern edges and predation levels yielded minimal effects. It may well be that various colour pattern statistics differ in importance relative to different functions and conditions.

Detectability is also influenced by how colour patches deviate from the spatial frequency distribution of the viewing background [2,28]. This may result in different components attracting attention at different distances [2,56]. Moreover, tannins and other pigments in the water and the colour of the spacelight will cause some colours to blend with the background, whereas others will remain conspicuous [11,35]. Lighting conditions must be considered carefully [11] because detecting the colour of individual colour pattern elements becomes more difficult as patch sizes decrease, viewing distance increases and light intensity decreases [57]. Endler formally tested the importance of background matching in shaping colour pattern evolution by lining his artificial streams with either fine or coarse multi-coloured gravel and manipulating the background against which predators perceived guppies [28]. When predators were present, larger gravel was associated with the evolution of significantly larger male spots, whereas smaller gravel favoured smaller spots. The reverse was true when predators were absent (fig. 3 in [28]), suggesting that background matching influences predation outcomes.

### (c) Structural colour visibility

Endler [2] observed that predators are most likely to detect male guppies via their highly reflective structural colours, which generate intense ‘flashes’ of colour visible over long distances (as in other taxa [58]). Guppies produce such colours via arrangements of micro-platelets situated in iridophores [10] that scatter light of select wavebands across a restricted angular range and are highly efficient reflectors. The visibility of guppy structural colours depends on viewing and illumination angles. In the shallow water of guppy habitats, illumination comes from above in a relatively narrow angle called Snell's window [59], which can enhance the effects of structural colours since reflectivity is strongly dependent upon illumination and viewing angles [57–59]. Structural colours in guppies also vary subtly in colour (hue) depending upon the precise orientation from which they are viewed and illuminated; the same flank region may appear greenish-blue, teal or bluish-green when seen from different angles. Many structural colours reflect in the ultraviolet, and this often underlies the pigmentary red/orange/yellow spots that occur across the guppy flank [24,25] (fig. 1 in [15]). The structural ultraviolet (UV) may serve to visually amplify [60] these longer wave pigmentary colours [10,61], or vice-versa [62]. More generally, the coincidence of orange pigment and structural UV means that such colour patches are visually dynamic; their apparent hue or luminosity may shift markedly with even subtle changes in viewing angle or when the guppy moves between shade (no UV) and direct sunlight (UV rich). This variation is enhanced by how male displays involve rotation of the body axis, which induces fluctuations in colour, intensity and shape.

The features that make structural colours visually dynamic make them challenging to quantify. Their restricted angular visibility, for example, means they cannot be effectively measured using a fixed photography or spectrometry configuration. This is because different individuals can vary in the orientation at which their structural coloration is visible. Simply photographing all guppies at a single ‘standard’ orientation will likely underestimate structural reflectance across fish to varying degrees depending on how far the set-up displaces each individual from its optimal camera viewing orientation. Irrespective of the recording design, illumination should come dorsally, as from Snell's window, and the camera or sensor should be positioned to emulate the orientation from which females or predators naturally view males. Predator versus conspecific viewing angles can be different and should be accommodated in any study of animal coloration.

Given that structural colours arise from selective reflectance (as opposed to selective absorption), their visual effect is often accentuated by the co-occurrence of pigments [61,62]. Underlying layers of melanin pigment, for example, absorb broadband light which would otherwise reflect from or transmit through the signalling surface, thereby acting in the manner of how a blackboard gives visibility to chalk. Guppy structural colours are unusual because they extend across largely unpigmented male flank regions. The suffusion of iridescent violets, blues and greens on the posterior flank and caudal peduncle is often exclusive rather than inclusive of the black spots and dynamic fuzzy black markings that occur in this region (figure 1). This makes for potentially high within-pattern contrast, yet the degree of such contrast depends on both the angle at which the flank is viewed as well as the immediate viewing background. Structural colours will be most obvious when minimal light is propagated from behind, that is, when an individual is viewed against a dark background (figure 1) such as the water column [20]. Such colours will engender the most startling visual effects when a male is lit by a narrow shaft of sunlight, which might often occur under dappled forest light. This is especially true when low solar angles predominate early or late in the day (when male guppies are more likely to be courting females [7]). These scenarios enable courting males to present their structurally coloured phenotype to the greatest effect but are also when they are most vulnerable to long-range detection by predators (*sensu* [2,28,29]), although there are ways around this [20]. It is critical to emulate this visual geometry to faithfully account for the biological consequence of structural coloration [57].

Endler [2] addressed these problems using multiple assessment methods. He first observed unanaesthetized fish in natural daylight, which means that he saw them from different angles and against different backgrounds as they moved and was able to record the presence of all structural colours. He then photographed all fish to measure spot size. Subsequent research on guppy coloration (e.g. [15,16,23,36,44,48]) is based near entirely on data from photographs taken against white backgrounds. This will systematically underestimate the occurrence of structural colour and, by extension, its potential visual interaction with pigmentary markings. A relatively simple way to enhance the visualization of structural colour is to photograph the fish on both white and black backgrounds while ensuring that the geometry and lighting and camera/sensor replicates what female guppies see in the wild. White provides a good background for measuring pigment-based markings whereas black is more effective in highlighting structural colour [14] (figure 1); guppies are seen against both kinds of backgrounds in nature [20].

The potential danger of inadequate structural coloration assessment is illustrated by male guppies from the Guanapo River and its tributaries, including the El Cedro River [25]. Males from these and other Trinidadian locations display pronounced structural UV, violet, blue and green markings. As noted earlier, adaptation under experimentally relaxed predation risk in the El Cedro River and four other Guanapo tributaries resulted in the enhancement of structural colours at the expense of pigmentary colours [14,25], which is fundamentally different from the evolutionary response observed by Endler in his Aripo experiment [28]. Failing to account for such coloration has led past authors [15,16,23] to conclude that adaptation to low predation generates less colourful ornamentation.

### (d) Viewer sensory properties

Correct inferences about the selective effects of predation demand an accurate representation of the presence and relative abundance of different predators across focal study sites [2]. This is particularly important when sensory-based modelling is employed to estimate prey visual conspicuousness. Considering relevant observers is important because different predator species vary in visual properties [11]—including spectral sensitivity and spatial acuity—but also because of differences in behaviour and ecology that determine the visual context under which they operate. For example, locations with only *Rivulus* or prawns tend to have closed or nearly closed canopies and a different set of light conditions to the more exposed stream habitats where *Crenicichla* are typically found, although canopy closure varies even within predator locations [14]. Diurnal predators will obviously differ from nocturnal predators in how they select prey coloration. Likewise, different predators may differ in their characteristic attack range (see §2b). Despite these qualifications, there is a significant tendency for loose assumptions regarding the natural history context of prey colour evolution, including substituting proxies for predatory species when information is otherwise lacking [4,42]. We detail below how this tendency is evident in the recent tests of parallel evolution in guppies [15,16].

Endler conducted timed visual censuses of the predator community at all sampling sites so that each site could be associated with a list of the species of diurnal predators and their relative abundance [2]. He also quantified the attack rate of the different predatory species and then developed an index to estimate the severity of each guppy predator species. He found a gradient of predation as one progressed from downstream to upstream. The total number of fish species and the number and abundance of species that prey on guppies is greatest downstream and progressively declines upstream. For example, *C. frenata* (the most dangerous guppy predator) was also one of the species most likely to have its upstream distribution stopped by natural barriers like waterfalls. This gradient in predator communities was matched by a gradient of male coloration, as assessed by variables such as spot number and spot size across the different colour categories (tab. 3 and figs 11–13 in [2]).

By contrast, the studies by Yong *et al.* [15] and Valvo * et al.* [16] report no assessments of predation and instead classify the communities dichotomously as either low or high predation risk. This is problematic because the composition of the community will affect the expected magnitude of the selective differences between their high and low predation localities. The dichotomous treatment of predator communities was first associated with research on life-history evolution [63], where it was based on a deliberate restriction of study sites to those that either had the full complement of potential predators versus those where guppies co-occurred with only *R. hartii*, which feeds primarily on invertebrates but will also prey opportunistically on vertebrate prey, including guppies [64–66]. Subsequent work has revealed a gradient of life-history traits, like fecundity, that matches the gradient in predator communities [67] and coloration [2].

Some Trinidad river communities have changed dramatically in the 45 years since the work by Endler. In fact, changes were even noted in the late 1980s [68]. The Guanapo, for example, now has a large, active quarry that deposits abundant sediments in the river. Ehlman *et al.* [69] replicated prior census techniques [65] to show that *Crenicichla* abundance has declined dramatically in the Guanapo over the past 25 years. The Guanapo high predation locality was included in the studies of Yong *et al.* [15] and Valvo *et al.* [16]. Given the known rate at which male colour patterns can evolve [28], it is conceivable that the differences among guppies from low and high predation communities now may be different from what they were during Endler's studies.

Yong *et al.* [15] and Valvo *et al.* [16] include sites on the north slope of the northern Trinidad mountain range, where there is a different community of predators than exist throughout the drainages on the south slope. Rivers on the south slope were once tributaries of South American Rivers and are dominated by cichlids and characins. Rivers on the north slope are dominated by fish with a marine origin; cichlids and characins are almost always absent. The primary predators are Gobies (*Gobiomorus*, *Dormitator*), mountain mullet (*Agonostomus*) and the diurnal freshwater prawn *Macrobrachium crenulatum*, which has very different colour vision from fish [2]. There are no published assessments of the fish communities of the north slope rivers comparable to those of the south slope, so the distribution and abundance of the key predators is poorly characterized (but see [2] and [68]). Many reports of life histories and male coloration that are inconsistent with the expected differences between high and low predation communities (e.g. [23,70]) are based on work done on the north slope rivers without the benefit of any attempt to assess abundance, distribution or predator risk. It is essential to assess causal factors directly and accurately.

### (e) Multivariate selection and genetic response

In studies of complex multi-trait phenotypes like guppy colour patterns, it is unreasonable to assume that selection will influence all components of male colour patterns in similar ways across populations. There are three main reasons: selective factors differ among traits, the form of the selection variance-covariance matrix among the traits and the form of the genetic variance-covariance matrix.

Individual colour pattern components are targeted differently by different selective factors [53]. This might explain why patch boundaries measured using boundary strength analysis in combination with visual acuity modelling and automated image segmentation are important in a study of guppy mate choice [41] but apparently not in a study looking at predation risk that uses the same boundary strength pattern statistics but without acuity modelling and with human subjective image segmentation [15]. One possible explanation for this is that guppy courtship takes place close enough for very fine detail to be seen [39], but all predators attack from a much longer distance and have lower acuity and more restricted colour vision than guppies [11], as in figure 2*b*. With greater distance and lower acuity, either boundaries would blur or the differences on either side of the blurred boundary would be greatly reduced, rendering boundaries less salient to predators or generally reduce overall conspicuous. Predators would still perceive larger patches and colour contrast, even if reduced compared to what female guppies see.

Each male colour trait will be selected in different ways depending upon the viewer and visual conditions under which the colour pattern is seen [11]. Hence, differences in female preference among populations will engender a different selective profile. Variation among female preferences is expected under sexual selection theory even under identical conditions because the Fisher process can run in multiple directions under identical conditions even with one mating and one trait locus [51]. Likewise, predators that possess different visual systems will induce natural selection for crypsis in different ways causing variation in selection as a function of geographical variation in predator communities. The consequence of such variation can be seen in guppies from populations that coexist with predatory prawns, which are unique among guppy predators in their ability to see in the ultraviolet but not in the red-orange range of the spectrum [2]. Male guppies in these populations have smaller and less reflective ultraviolet markings but more extensive orange [24,44], as expected if such traits were selected through the prawn visual system. Additional local factors such as canopy openness, ambient light colour [20], water clarity, water colour and background type [20] may bias colour perception in site-specific ways (e.g. [14]). Each trait is therefore likely to have a different array of natural and sexual selection effects, ‘targets’ and selection directions [38,71]. Some traits will evolve due to sexual selection and not predation, others will evolve due to predation and not sexual selection, and others will evolve in response to both but to varying degrees depending upon the general biology, physics and the habitat variation among and within streams. Moreover, the same traits may respond differently to different viewers [11] and motion contexts (figure 2). It is important to investigate which colour pattern components are subject to which modes of selection.

There is an unfortunate implicit assumption in the literature that selection is the same for all components of a colour pattern. This ignores the multivariate effects of selection, particularly the effects of correlational selection, where selection on one trait may be positively or negatively correlated with selection on another trait [34]. Correlational selection is summarized by the selection variance-covariance matrix (*γ*) which also includes the effects of nonlinear (disruptive or stabilizing) selection [72]. These effects are ignored in most studies but are potentially important because they can affect evolutionary trajectories [34] in unexpected ways, especially when *γ* contains both positive and negative covariances and varies among populations. The action of *γ* can also cause the genetic architecture to evolve in particular directions, especially if there are negative selective covariances [34]. Further evolutionary constraints can be caused by stabilizing or disruptive selection as estimated by *γ* [34,72].

Even if there were no selective covariances or nonlinear selection [72], multivariate evolution can be influenced by genetic architecture (summarized by the genetic variance-covariance matrix, G). If some traits are negatively genetically correlated, then selection on one trait can cause opposite changes in another trait, and, if positively correlated, selection on one could yield changes in another even if not selected. Moreover, the form of *γ* can affect the evolution of G, further affecting the course of evolution. Variation will be generated by among-population differences in either or both *γ* or G, neither of which have been investigated in guppies. Generally, phenotypic selective and genetic associations among traits may either facilitate or impede adaptation. Genetic architecture should have a major effect on how colour patterns evolve and diverge, meaning that a multivariate trait approach should be central in efforts to understand parallel evolution [73].

Consideration of guppy colour patterns as multivariate traits suggests that study conclusions will be highly dependent upon which traits are included in the estimation of colour phenotype (as in §2c). It also poses major problems for studies such as [15] and [16] that seek to dichotomize sites as simply ‘high’ and ‘low’ predation risk. Predation risk is a quantitative phenomenon that will induce different modes of natural selection upon different traits that constitute the overall colour pattern. Moreover, parallel evolution is manifest in a range of intensities owing to the common appearance of within-environment divergence [32], so we must be cautious in interpreting statistical tests for parallelism. We cannot regard parallel evolution as absent or present, especially when there is a diversity of evolutionary trajectories within a given environment [20], as in guppies [2,27,35].

## 3. How do different methods compare?

**Figure 3.**
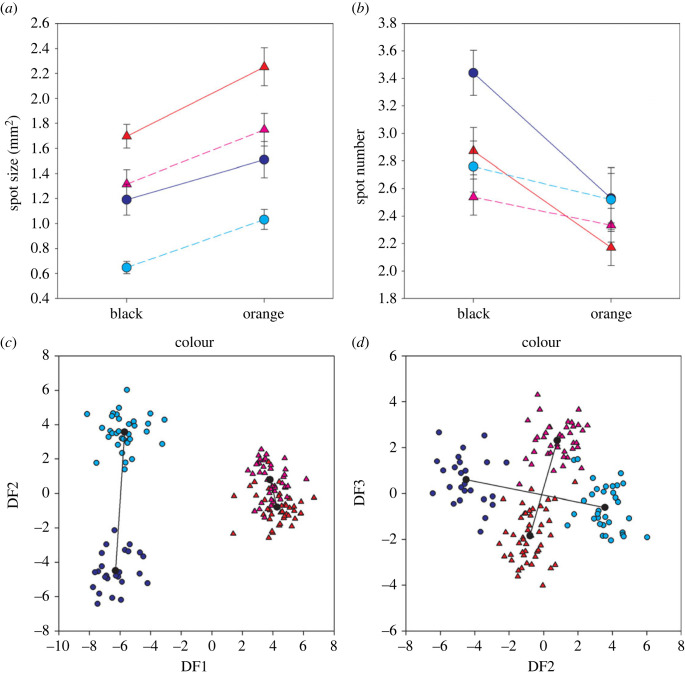
Analysis of a high-predation and low-predation population each from Aripo and Arima rivers. (*a*) Average (mm, ± 1 s.e.) black and orange spot area. (*b*) Average (± 1 s.e.) spot number. Low-predation populations had larger spots of both colours and more black spots (*p* < 0.001 for each) in both rivers but neither showed a difference in orange spot number (*p* = 0.09). We also conducted a discriminant analysis of principal components (DAPC) on colourmesh data on the same fish. (*c*) DF1 and DF2 with DF1 differentiating among rivers and DF2 between Arima predation regimes. (*d*) DF2 and DF3, the latter differentiates among predation regime in the Aripo river.

New approaches to colour pattern analysis challenges the field to reconcile results gained via different methodologies [30]. For example, both Yong *et al.* [15] and Valvo *et al.* [16] conclude that their results differ fundamentally from Endler's, yet they reached this conclusion via different methods or accounted for different traits than Endler's [28]. It is therefore appropriate to ask how their and Endler's results compare if performed on the same data. We report an application of Endler's and Valvo's *et al.* methodologies to the same series of Endler's original photographs of males from high/low predation environments on the Aripo and Arima Rivers [2]. These date from before the existence of calibrated cameras but did have colour standards in them. The high predation environments were ones that had the full spectrum of diurnal predators (*C. alta, Aequidens pulcher, Hoplias malabaricus, Astyanax bimaculatus, Hemibrycon dentatum*). The low predation sites had only the killifish *R. hartii*. Because we were working with photographs of fish on a white background, we only enumerated the number and size of orange and black spots (see §2c). Our results for the Endler analysis parallel his original results, which are that the representative males from low predation environments have larger orange and black spots and more black spots than their counterparts from high predation environments downstream ([2]; figure 3*a,b*). Neither we nor Endler [2] found more orange spots in low predation environment. Overall, the differences support Endler's original conclusion that males from low predation environments are more highly ornamented than those from high predation environments in the same stream. Note that this test is only a partial assessment of male coloration because we were not able to characterize structural colour from these photographs, and neither Endler's original studies nor our analysis assessed patch boundary strength, which is what Yong *et al.* evaluate.

We applied Valvo's *et al.* methods [16] to this same set of photographs. As in [16], we found that one discriminant function discriminates among rivers. However, we did not find a single function that discriminated between high and low predation (figure 3*c,d*), therefore questioning the dichotomous classification of predation regimes. In addition, we performed their bootstrap analysis for parallelism using the R-code they provided and found that the distribution of vector correlations is slightly anti-parallel (mean −0.093, CI −0.0879 to −0.0978). That is, changes in the two rivers are, if anything, divergent.

How do we resolve this apparent contradiction in results and conclusions? First, Endler and Valvo *et al.* (and Yong *et al.*) measured very different traits. Endler measured spot size and number grouped by colour class using manual image segmentation assuming high acuity [39]. Valvo *et al.* measured the colour of specific pixels across the body without any image segmentation or consideration of predator perception. Although spot size and number will be correlated with the number of differently coloured pixels, simply counting pixel abundances does not capture pattern (as per the approaches by Endler and Yong *et al.*). Second, the two methods have different expectations and interpretations. Endler tested for a change in spot size and spot number *on average* between different predation regimes, while Valvo *et al.* (and Yong *et al.*) tested if the high-low predation magnitude was the same in each pair of streams. Endler's interpretation of increased ornamentation in low-predation versus high-predation regimes is agnostic on the specific changes (i.e. which colours) precisely because this was already known to vary among streams (as implied in fig. 7 of [2] and made explicit in [35]). Given this, the remarkable finding is not that Valvo *et al.* failed to find parallel changes in four of their six pair-wise comparisons but that they did find a significant correlation in two cases (the El Cedro-Guanapo and Aripo-Turure). Again, this is partially due to measuring very different traits with very different effects on guppy versus predator vision and perception. It is not surprising that predators do not select for increased boundary contrast [15], given that effects of visual acuity, attack distance, and water transmission may eliminate or reduce boundary contrast. By contrast, guppies can see all aspects of colour patterns in fine detail [20,39].

These results, and our five more general comments mentioned above, provide critical guidance on how to design and execute further research on this system. First one needs a specific hypothesis. Endler's approach has been successfully tested with an array of experiments on female choice [7,11,13,14,22,24–26,28,29,35,36,38,39] and predation [2,11,14,24,25,28,29], and well-replicated experimental studies of evolution in natural and artificial streams [28,29,39]. Nevertheless, we need to constantly improve the breadth of traits measured while ensuring all biologically relevant conditions are measured and accounted for. We need to learn more about how female guppies and predators process visual information A logical first step is to do what we did here, which is to compare the results when different methods are applied to the same data. Here, we show that Endler's original results are repeatable and not well correlated with those of Valvo *et al.* Such contradicting findings emphasize the importance of understanding the implications of using different methods in quantifying animal coloration and how using different methods affects our theoretical understanding and the literature. A second step would be to perform more detailed behavioural choice assays with female guppies [41], predation assays [2] and joint effects of variation in the light environment and visual backgrounds [20] to explore and predict correlations between animal behaviour and as many colour pattern parameters as possible and to investigate the differing functions of different colour pattern components (e.g. [30,41,53]).

## 4. General factors affecting the function and evolution of colour patterns

We have identified pitfalls in guppy coloration research, but this gives only a partial picture of all the factors affecting the function and evolution of colour patterns. Consequently, we provide a list of multiple factors in table 1 which need to be seriously considered when studying colour patterns of any species. These factors are organized in the order in which light travels from the sun, environment and visual target through the eye to the brain and decision-making. Further discussion is found in [2–4,6,59]. This is not an exhaustive list, but it provides a framework for future detailed study of colour patterns. There is significant variation in the degree to which each item in this list has been investigated, and we hope it will encourage research into neglected aspects of how we understand colour patterns. By analogy, a similar set of factors will affect other sensory modes.

**Table 1.** Important considerations for analysis and conclusions based upon coloration. Each heading is a process which depends upon the items in the list. The last line or lines provide some consequences. Further details in [4,6,59].

ambient light spectrum and intensity
season and time of day
relative amounts of sunlight and clouds
canopy closure, cloud cover and clouds over sun or not
affects all aspects of vision
reflection off both the average and current visual background
what is in the field of view
ambient light spectrum on background
spatial variation within the background
affects chromatic and luminance light adaptation hence coloration perception
reflection off the guppy (or other target animal) and transmission
ambient light striking the guppy
reflectance spectrum from each colour pattern component
the angle of structural colours relative to ambient light and viewer
whether light is direct or diffuse, depends upon cloud cover
spectral attenuation by the water between the guppy and viewer (distance dependent)
with ambient light, determines spectrum, intensity and direction of guppy-viewer radiance
affects reception and perception of colour patterns
phenology of viewer and viewed
time of day and microhabitat during which courtship or social activities occur
time of day and microhabitats during maximum and average predation risk
light spectrum and intensity changes with time of day and weather directly affecting visibility of colour patterns
reception by viewer
viewer's attention captured by the guppy (viewing correct direction)
ambient light striking the guppy
distance and angle to guppy
relative spectra of guppy and visual background
relative complexity of guppy and visual background
spectral composition of light coming from viewers field of view and guppy
the resolving power (acuity) of viewer
the viewer's flicker fusion rate (ability to see moving objects)
the guppy's speed in the viewer's field of view
the geometry of the guppy's motion
the within-guppy visual contrast
the guppy-background visual contrast (dynamic)
affects what reaches the viewer's eyes
viewer species can vary extensively in visual and other sensory abilities
retinal processing and detection by the viewer
spectral sensitivity of viewer's photoreceptors
relative abundance of viewer's photoreceptor classes
chromatic adaptation of viewer's photoreceptors
the photoreceptors reaction times (photopigment regeneration rate) of viewer visual neural circuitry
susceptibility to sensory biases
spatial and temporal patterns of variation in retinal processing resulting from
variation in environment, background and motion of both viewer and viewed
different for each viewer, even within species
perception and discrimination by the viewer
viewer's attention captured by the guppy
cognitive processes including colour classification and categorization,
use to which colour pattern information is put, previous experience with coloration, etc.
within-guppy and guppy-background visual contrasts and their relative values
likely to vary among viewers, both among and within species.
likely to vary within a viewer, depending upon viewer's physiological state and experience
preferences and decision making by the viewer
viewer's attention held long enough to make a decision about the guppy
viewer's inherent preferences, usual use of colour patterns and colour-based decisions
current and previous experience with consequences of choices based upon coloration
likely to vary among viewers, both among and within species.
likely to vary within a viewer, depending upon viewer's physiological state and experience
guppy (or other prey) fitness consequences
which predator species are present and which species are most risky
local mean and variation in female preferences
environmental conditions including environmental changes at all-time scales
can be modified by guppy behaviour such as microhabitat choice and phenology

## 5. Conclusion

The first studies of ornament evolution in guppies [2,28,29] set high standards in terms of the number of localities sampled, the characterization of the fish community, guppy risks of predation and the multifaceted approach to quantifying colour patterns. Significant innovations have arisen since, including techniques to better characterize structural colour [14,24,25], greater insight into the visual capacities of guppies and their predators [8,12] and an increased understanding of how guppies behave in the wild [20]. In this paper, we raise concerns aimed not at the new analytical tools themselves but at the way they should be applied or are used to derive biological conclusions. Investigations of animal coloration using different tools to investigate similar topics demand close scrutiny. In our case, two studies [15,16] employing very different methodologies conclude that their results are at odds with Endler's conclusions. Our critical appraisal of these two studies identifies emerging misconceptions about parallel evolution and foreshadows the issues that will accompany the application of new colour analytics. The major problem is conflating parallel evolution with adaptive evolution; sexual selection and strongly polymorphic traits negate that implicit assumption.

The five points we outlined above offer manifold reasons why the studies of Yong *et al.* and Valvo *et al.* neither dispute Endler's original results nor reasonably address the hypothesis of parallel evolution. Instead, it could be argued that, just like Endler's work, both studies rely upon a specific way of measuring colour patterns with substantially varying degrees of considering structural colour in long-range detection by predators [2], variation among localities in how such colours are selected by females [35,38] and the composition of predator communities. Different methods address different aspects of function. Given the passage of time and the known changes in habitats [69], it is necessary to reassess the predator communities.

More broadly, guppy colour patterns comprise multi-trait phenotypes which will evolve according to multivariate selection in ways prescribed by genetic architecture and subject to the diversifying and multivariate effects of sexual selection. These processes generate complexity in how the colour pattern will evolve, even under comparable selection intensities. The contribution of sexual selection adds to the diversity of evolutionary outcomes because female guppies from different populations differ in their colour preferences and sexual selection favours this divergence [35,38].

It is important to note that none of Endler's [2,7,11,20,28,29,35,36,38,39,63] or colleagues [9,13,14,24,25,27,50] work on male coloration in guppies mentions parallel evolution. Instead, this work has focused on how the correlations and trade-offs between factors influencing natural and sexual selection shape observed guppy coloration. Expectations for evolutionary parallelism among populations subject to relaxed predation risk should not be for male colour patterns to evolve similarly, but according to the vectors initially favoured by females in each population and varying visual conditions. Studies of parallel evolution frequently find considerable variation within a selective regime [32,39], and there are various degrees of parallelism, which means that treating it as a yes/no conclusion is unrealistic [32]. Furthermore, the absence of parallelism does not mean an absence of adaptive evolution; one does not necessarily lead to the other. That should come as no surprise when dealing with the combined effects of sexual and natural selection on a multi-trait phenotype.

For colour patterns exhibiting extensive polymorphism and subject to sexual selection, we predict no or very weak parallel evolution because there are a large number of equally fit optima. However, we do predict parallel evolution for traits which are not subject to sexual selection and have single population specific optima based upon physics and physiology, such as guppy life-history traits. There is no reason to expect that all traits of a species should evolve in the same ways.

Finally, there remains a significant challenge to understand how the varied methods for quantifying colour patterns and within them the diversity of parameters predict how they are perceived by ecologically relevant viewing audiences. Endler recognized the potential limitations of only considering colour patches themselves, rather than the boundaries between them which is why he suggested boundary analysis [71], which was expanded in QCPA [30] and used by [15] and [41]. QCPA allows a very large number of different pattern measurements to be made [30]. This leads to the problem of how to decide which parameters are biologically relevant. This is made more complicated by the fact that different parameters may be the targets of different kinds of natural and sexual selection. Choice of the best parameters to predict behaviour and evolution will depend upon a detailed knowledge of ecology, neurobiology and behaviour. The collective efforts to understand how and why male coloration has evolved in guppies indicate the challenges associated with studying colour evolution more generally (table 1).

## Data Availability

Data are available in Dryad Digital Repository with the DOI: https://doi.org/10.5061/dryad.qfttdz0ns [74]. Supplementary information is provided in electronic supplementary material [75].
